# Investigating the microbiome of house dust mites in South Korea

**DOI:** 10.3389/falgy.2023.1240727

**Published:** 2023-08-16

**Authors:** Myung-hee Yi, Myungjun Kim, Tai-Soon Yong, Ju Yeong Kim

**Affiliations:** Department of Tropical Medicine, Institute of Tropical Medicine, Arthropods of Medical Importance Resource Bank, Yonsei University College of Medicine, Seoul, Republic of Korea

**Keywords:** house dust mites, microbiome, *Dermatophagoides farinae*, 16S rDNA, *Bartonella*, *Enterococcus*

## Abstract

Understanding the house dust mites (HDMs) microbiome is crucial due to its potential effects on the development of allergic diseases. In 1998, our laboratory collected *Dermatophagoides farinae* and *D. pteronyssinus* from beds in a Korean household and began cultivating these HDMs. Our laboratory has been actively investigating several topics about HDMs in recent years, including the bacterial and fungal microbiome and their interactions, as well as the impact of the HDM microbiome on airway inflammation. To study the *D. farinae* microbiome, we employed high-throughput sequencing of the 16S rDNA amplicons. The results revealed that the two most abundant bacteria were *Enterococcus faecalis* and *Bartonella* spp. In contrast, we found almost no bacteria in *D. pteronyssinus*. By inoculating bacteria to HDMs, we found that *D. farinae* is more susceptible to bacteria than *D. pteronyssinus.* This susceptibility was associated with the presence of certain fungal species in *D. pteronyssinus*. Additionally, we have recently made efforts to produce HDMs with reduced levels of symbiotic bacteria. We believe that standardizing and controlling the microbiome in HDMs are crucial steps for the future development and improvement of allergic immunotherapies.

## Introduction

House dust mites (HDMs) are widely recognized as the primary sources of indoor allergens, playing a crucial role in triggering allergic diseases such as allergic rhinitis, asthma, and atopic dermatitis (1–3). Approximately 50% of individuals with asthma have an allergic reaction to HDM (4). More than 30 specific HDM allergens have been identified as key triggers in allergic responses (5). Professor Ree in our department identified 23 species of mites from house dusts in South Korea in 1997 of which *Dermatophagoides farinae* and *Dermatophagoides pteronyssinus* were reported to be the most predominant HDM species (6). The predominant species of mites found in Korean households were determined to be *D. farinae*, present in 65%–77% of homes, and *D. pteronyssinus*, found in 8%–20% of homes (6, 7). HDMs are present in over 90% of households in Korea, and the degree of exposure to HDM is medically noteworthy (8). Approximately 40%–60% of Korean individuals with respiratory allergies are affected by HDM (9–12), and more than 40% of patients with atopic dermatitis exhibit sensitivity to HDM (13–19).

Afterward, in 1998, our research lab collected *D. farinae* and *D. pteronyssinus* from beds in a Korean household and cultivated these HDMs. We have been continuously cultivating them and conducting research on HDM allergens and allergic diseases up to the present. Furthermore, our laboratory also serves as a resource bank, where we cultivate various mite and cockroach species and supply these to other institutions for their research needs. In our laboratory, we have been actively investigating the following topics using HDM in recent years: bacterial and fungal microbiome and their interaction, and the impact of the HDM microbiome on airway inflammation. Therefore, in this manuscript, we would like to provide an overview of our lab's recent research.

Immune responses are modulated by the properties of the allergen itself and by the adjuvant-like substances that are concomitantly administered with the antigens. Characterizing the HDMs microbiome is crucial due to its potential immunomodulatory effects on allergic disease pathogenesis, through the generation of microbe-associated molecules such as lipopolysaccharides (LPS), lipoteichoic acid, and bacterial DNA (20, 21).

## Microbiomes of the HDMs

**Figure 1:**
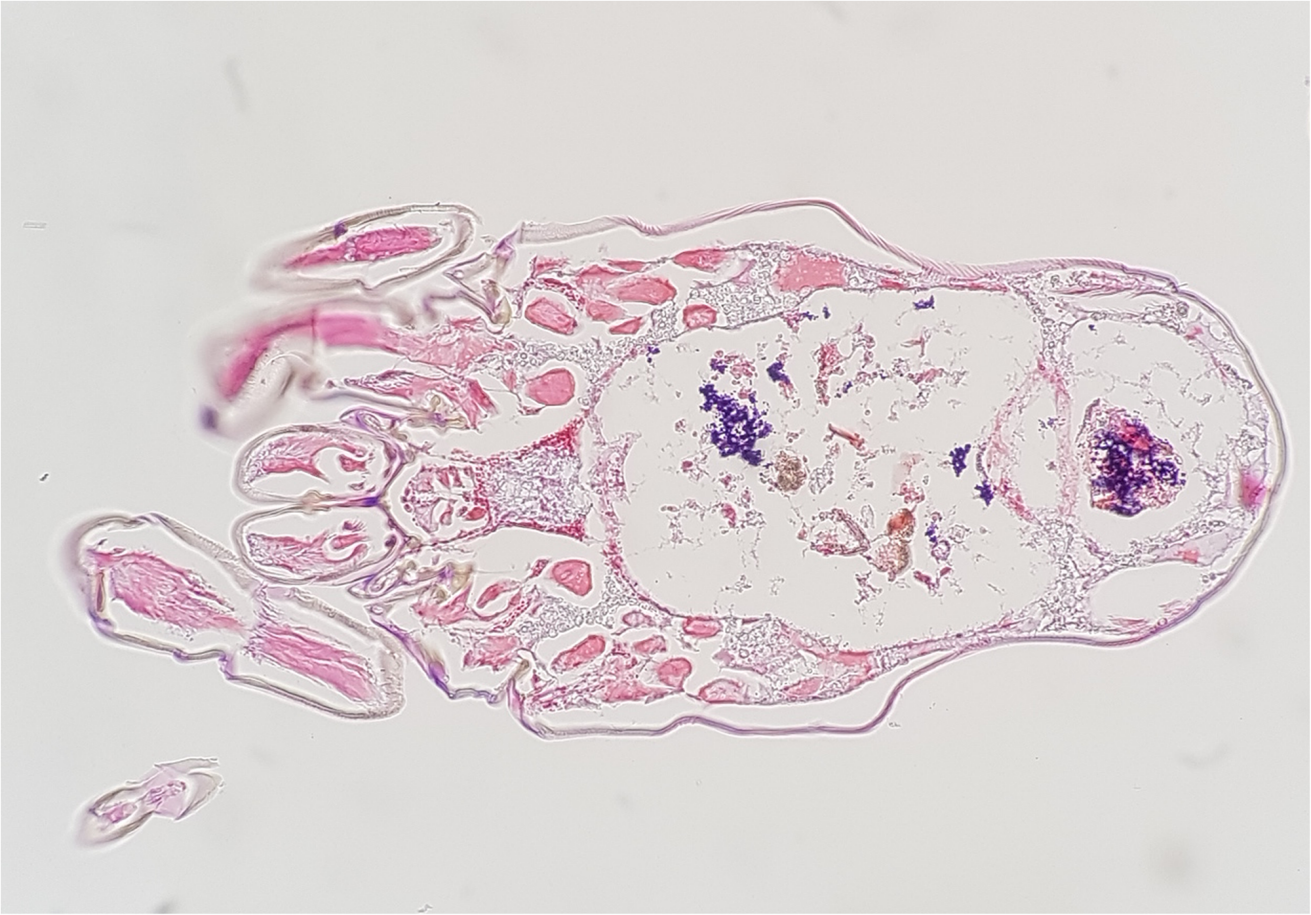
*Enterococcus faecalis* (gram-positive cocci, stained blue) observed in the intestine and stool of *dermatophaoides farinae*, Gram staining (magnification ×200).

In 2018 we studied the *D. farinae* microbiome using the 16S rRNA amplicon deep sequencing (22). In this study, 113 bacterial species were found, and the most abundant bacteria was *Enterococcus faecalis* (53.6%) and the second most abundant bacteria was *Bartonella* HQ806746 (39.0%). We confirmed these results using the *E. coil* TA cloning vector system targeting the full sequence of 16S rDNA in mite's DNA. In addition, we found that *E. faecalis* were distributed throughout the intestine and the stool using Gram staining, and *Bartonella* spp. was detected in the hemocoel using silver staining (Figure 1).

In the next study, we investigated the bacterial microbiomes of *D. farinae* and *D. pteronyssinus*, both of which have been continuously cultured and maintained in our laboratory for over 20 years (23). It was found that these mite species have distinctly different microbiomes. As per our previous study, in *D. farinae* two dominant bacteria: *Bartonella* and *Enterococcus faecalis* accounted for 99.51%. In contrast, the bacterial sequence read was almost not detected in *D. pteronyssinus* except for several *Klebsiella pneumonia* reads. In quantitative PCR, *D. pteronyssinus* was found to have 600 times less total bacterial DNA than *D. farinae*. Interestingly, we found that the *D. farinae* collected from a house in Japan, which had been maintained in our laboratory for two decades, had the same *Bartonella* species as the Korean *D. farinae*. Thus, when bred in the same environment, mites of the same species showed almost identical microbiome patterns regardless of their regional origin.

There have been reports that *D. farinae* extracts produced by different countries and companies may have varying levels of included LPS, with differences of up to 9-fold (24, 25). This can be attributed to the variations in the methods employed by each company in breeding HDMs, which in turn can lead to differences in the microbiome of the mites. In fact, the microbiomes within HDMs vary across countries and institutions. The studies on HDMs microbiomes in the world were summarized in Table 1. A study of the draft genome of *D. farinae* in Hong Kong, China reported *Enterobacter* spp. was the most predominant (63.5%) and *Bartonella* spp. accounted for only 1.7% (27). *Staphylococcus*, *Escherichia*, *Rhizobium*, *Klebsiella*, *Endophytic*, *Bacillus*, and *Salmonella* were found in this study too in *D. farinae* (27). While another study using 16S rRNA cloning in the US reported that *Bartonella* was the most abundant bacterial taxon (20). In another study using similar methods in Czech, many bacteria were found including Sphingobacteriales, Rhizobiales, Neisseriales, Enterobacteriales, Pseudomonadales, Bacillales, Lactobacillales, Clostridiales, and Actinomycetales, but *Bartonella* was not detected (26, 33). Among these *Enterococcus* spp. was abundant bacterial species in *D. farinae* (26). In 2020 in China, a study using the 16S rRNA gene metabarcoding, the same methods we used, reported that *Klebsiella*, *Acinetobacter*, *Raoultella*, *Aeromonas*, *Streptococcus*, *Lactococcus*, and Escherichia were found in *D. farinae* (28). According to a recent 2020 study conducted in the Czech Republic, there is an association between *Cardinium* and *D. farinae*, indicating that *Cardinium* might have biological functions within the *D. farinae* (29). Other bacteria found in this study were *Cloacibacterium*, *Streptococcus termophilus*, *Streptococcus dentisani*, *Dolosigranulum pigrum*, *Lactobacillus fermentum*, *Staphylococcus saprophyticus*, *Bacillus anthracis*, *Propionibacterium acnes*, *Corynebacterium tuberculostearicum*, *Lawsonella clevelandensis*, *Ottowia beijingensis*, *Curvibacter fontanus*, *Acinetobacter vivianii*, *Moraxella osloensis*, and *Alphaproteobacteria.* Another study in Czech in 2020, detected *Staphylococcus arlettae*, *Lactobacillus fermentum*, *Cardinium*, *Pantoea*, *Acinetobacter radioresistens*, and *Lactobacillus amylovorus.* In the study conducted in France in 2021, Bartonellaceae, Sphingomonadaceae, Enterobacteriaceae, Amoebophilaceae, Caulobacteraceae, Patubacteraceae, and Myxococcales were found in the *D. farinae*.

**Table 1:** Summary of studies on the microbiome of house dust mites (*Dermatophagoide farinae* and *D. pteronyssinus*).

Country (Year)	*Dermatophagoide* species	Major microbiome	Ref.
US (2005)	*D. farinae*, *D. pteronyssinus*	*Bartonella*, Gram-negative enterics	(20)
Czech (2012)	*D. farinae*	Sphingobacteriales, Rhizobiales, Neisseriales, Enterobacteriales, Pseudomonadales, Bacillales, Lactobacillales, Clostridiales, Actinomycetales	(26)
China (2015)	*D. farinae*	*Enterobacter*, *Staphylococcus*, *Escherichia*, *Rhizobium*, *Klebsiella*, *Bartonella*, *Endophytic*, *Bacillus*, *Salmonella*	(27)
Korea (2018)	*D. farinae*	*Enterococcus faecalis*, *Bartonella*	(22)
Korea (2019)	*D. farinae*	*Enterococcus faecalis*, *Bartonella*	(23)
Korea (2019)	*D. pteronyssinus*	*Klebsiella*	(23)
China (2020)	*D. farinae*	*Klebsiella*, *Acinetobacter*, *Raoultella*, *Aeromonas*, *Streptococcus*, *Lactococcus*, Escherichia/Shigella.	(28)
Czech (2020)	*D. farinae*	*Cardinium*, *Cloacibacterium*, *Streptococcus termophilus*, *Streptococcus dentisani*, *Dolosigranulum pigrum*, *Lactobacillus fermentum*, *Staphylococcus saprophyticus*, *Bacillus anthracis*, *Propionibacterium acnes*, *Corynebacterium tuberculostearicum*, *Lawsonella clevelandensis*, *Ottowia beijingensis*, *Curvibacter fontanus*, *Acinetobacter vivianii*, *Moraxella osloensis*, *Alphaproteobacteria*	(29)
Czech (2020)	*D. pteronyssinus*	*Streptococcus*, *Staphylococcus*, *Bacillus*, *Propionibacterium*, *Burkholderiales, Alphaproteobacteria*	(29)
Czech (2020)	*D. farinae,*	*Staphylococcus arlettae*, *Lactobacillus fermentum*, *Cardinium*, *Pantoea*, *Acinetobacter radioresistens*, *Lactobacillus amylovorus*	(30)
Czech (2020)	*D. pteronyssinus*	*Staphylococcus arlettae*, *Lactobacillus fermentum*, *Lactobacillus amylovorus*	(30)
France (2021)	*D. farinae*	Bartonellaceae (87.7%), Sphingomonadaceae, Enterobacteriaceae, Amoebophilaceae, Caulobacteraceae, Patubacteraceae, Myxococcales	(31)
Czech (2021)	*D. pteronyssinus*	*Kocuria*, *Staphylococcus*, *Virgibacillus*, *Lactobacillus*, *Burkholderia*, *Oceanobacillus*, *Lentibacillus*, *Brevibacterium*, *Staphylococcus*	(32)

Various bacteria were found in *D. pteronyssinus* including *Kocuria*, *Staphylococcus*, *Virgibacillus*, *Lactobacillus*, *Burkholderia*, *Oceanobacillus*, *Lentibacillus*, *Brevibacterium*, *Streptococcus*, *Bacillus*, and *Propionibacterium* (29, 32), but it is considered that the number of bacteria in *D. pteronyssinus* is far less than those in *D. farinae* (23)*.* The previous studies support the notion that *D. farinae* extracts contain higher concentrations of LPS compared to *D. pteronyssinus* extracts (20, 29, 34–39).

## Susceptibility of *D. farinae* and *D. pteronyssinus* to bacteria

In our previous study, we discovered that *D. farinae* contained significantly higher levels of bacterial microbiome compared to *D. pteronyssinus*, predominantly consisting of *E. faecalis* and *Bartonella* spp. (23). To better understand this phenomenon, *E. faecalis* carrying a gene for green fluorescence protein was introduced into both *D. farinae* and *D. pteronyssinus* (40). After a 10-day inoculation period, the colony-forming units (CFU) per mite were measured. The results showed that *D. farinae* had 7,950 CFU/mite, while *D. pteronyssinus* had 6.65 CFU/mite, which demonstrates that *D. farinae* can maintain over 1,000 times more *E. faecalis* compared to *D. pteronyssinus*. In the time series analysis, the amount of *E. faecalis* kept decreasing exponentially in *D. pteronyssinus* after inoculation, but the amount of *E. faecalis* was maintained in *D. farinae* from day 6. Therefore, these results indicate that *D. farinae* has a higher susceptibility to bacteria compared to *D. pteronyssinus*, suggesting that *D. pteronyssinus* may possess factors that make it less suitable for bacterial symbiosis or parasitism.

## Fungi inhibit bacteria in *D. pteronyssinus*

Transcriptomic analysis of airway epithelial cells (BEAS-2B) treated with *D. farinae* and *D. pteronyssinus* extracts revealed enrichment of the antibiotic metabolic process pathway in *D. pteronyssinus*-treated cells, not *D. farinae*-treated cells (40). We hypothesized that the reason for the enrichment of this antibiotic metabolic process could be due to the abundance of specific fungus in *D. pteronyssinus* and measured fungal gene levels in two HDM species. Fungal genes were found to be 1,300 times more abundant in *D. pteronyssinus* than in *D. farinae*, with *Aspergillus penicillioides* being the predominant species identified through mycobiome analysis using fungus-specific ITS2 amplicons. In our study, *A. penicillioides* and *Pencillium cinerascens* were exclusively detected in *D. pteronyssinus*, while they were not found in *D. farinae*.

Meanwhile, bacterial genes were 12,000 times more found in *D. farinae* than in *D. pteronyssinus* in the study*.* Then, we hypothesized that these fungi in *D. pteronyssinus* could inhibit bacterial growth in the mites. To prove this, *D. pteronyssinus* were inoculated with *E. faecalis* and treated with amphotericin B, the antifungal agent. After a 10-day period, it was observed that the CFU of *E. faecalis* per mite was 40 times higher in *D. pteronyssinus* treated with amphotericin B compared to untreated *D. pteronyssinus*. Furthermore, the extract of *A. penicillioides*, which was isolated from *D. pteronyssinus*, demonstrated inhibitory effects on the growth of *E. faecalis in vitro*.

*A. penicillioides* is a xerophilic fungus commonly found in low-moisture environments such as indoor air and house dust (41, 42). Its presence has been suggested to contribute to the reproduction of *D. pteronyssinus* (43). Notably, both *A. penicillioides* and *Penicillium cinerascens*, found in *D. pteronyssinus*, possess antibacterial properties (34–46). Similar to our study, *Saccharomyces cerevisiae*, *Aspergillus*, and *Candida* were reported in *D. pteronyssinus* in other studies (30, 32, 47, 48)*.* In addition, there have been several studies suggesting that the presence of fungi in insects can impact bacterial growth, which is consistent with our findings (49–51).

## Effect of microbiome of HDMs on airway allergy

To determine whether changes in the HDM microbiome affect allergy development, *D. farinae* were cultured under ampicillin and the number of bacteria in *D. farinae* was reduced by 25-fold (23). Moreover, the use of antibiotics led to a significant 100-fold reduction in the concentration of LPS. However, there was no difference observed in the concentration of the Der f 1 allergen in the HDM extract following antibiotic treatment. Notably, treating the BEAS-2B cells with the antibiotic-treated *D. farinae* extract resulted in a significant decrease in the secretion levels of proinflammatory cytokines IL-6 and IL-8. These findings indicate that alterations in the microbiome can impact the HDM's ability to trigger allergic diseases.

In addition, in the transcriptomic analysis of the response of airway epithelial cells to *D. farinae* and *D. pteronyssinus* extracts, we found that the “response to bacterium” and “pattern recognition receptor signaling pathway” were enriched in the cells treated with *D. farinae* compared with *D. pteronyssinus* (40). We were able to interpret that these results were due to the higher abundance of bacteria in *D. farinae* compared to *D. pteronyssinus*. *D. farinae* displayed a 14,000-fold higher bacterial DNA level, along with a 60-fold higher LPS concentration, compared to *D. pteronyssinus*. Treatment with an LPS inhibitor resulted in a decrease in the secretion of IL-6 and IL-8 in cells treated with *D. farinae* extracts but not in cells treated with *D. pteronyssinus* extracts. These results indicate that bacterial-derived substances such as LPS in HDMs have a synergistic impact on cytokine secretion in airway epithelial cells.

Many studies have reported a higher concentration of LPS in *D. farinae* compared to *D. pteronyssinus* (20, 29, 34–39). LPS plays a crucial role in triggering various immunological and allergic responses (52–55). LPS has been implicated in the development and severity of asthma (53, 56, 57). In a mouse model, depletion of LPS in HDM extract led to a reduction in features of allergic asthma (58). LPS can stimulate the development of type 2 immune responses to inhaled allergens through Toll-like receptor 4 (TLR4) signaling (59, 60). The HDM's LPS acts as a TLR4 activator in airway epithelial cells, leading to the induction of allergic inflammation through the activation of mucosal dendritic cells (55). Previous research demonstrated molecular-level differences in the lung transcriptome of mice challenged with *D. pteronyssinus* extracts containing high or low levels of LPS (61). HDMs trigger a type 2 immune response characterized by increased expression of IL-6 and IL-8 in airway epithelial cells (62, 63). Specifically, group 1 allergens and LPS from HDMs stimulate the expression of IL-6 and IL-8 by activating protease-activated receptor 2 (PAR-2) and TLR4, respectively (64). Furthermore, in a recent mouse asthma model, it was discovered that Gram-negative bacteria associated with HDM exacerbated the severity of the disease through nucleotide-binding oligomerization domain-containing protein (NOD) (31). Moreover, the presence of certain muropeptides from Gram-negative bacteria in the HDM extract potentially contributes to airway inflammation. Additionally, lipoteichoic acid has been found to influence the development of allergies through TLR2 (65). It has also been reported that LPS from *Bartonella* acts as an antagonist of TLR4 (66). Therefore, it is important to consider the composition of the microbiome within the mite to accurately evaluate the immunotherapeutic effectiveness of the extract.

## Production of *Dermatophagoides farinae* having low bacteria

As observed earlier, the microbiome of HDMs and their associated substances can have an impact on immune responses in humans. Furthermore, symbiotic bacteria within HDMs can exist as different species depending on the country or rearing environment. Hence, accurately predicting all the immunological side effects associated with each bacterium poses a significant challenge, particularly in the clinical use of immunotherapeutic agents.

We recently conducted a study where we treated *D. farinae* with ampicillin during their rearing process. We published our findings, demonstrating that despite multiple sub-culturing events, the bacterial levels in *D. farinae* remained low for 18 weeks following a single ampicillin treatment (67). In this study, we administered ampicillin at an appropriate concentration to ensure it did not affect the growth of HDMs. As a result, we successfully achieved a 150-fold reduction in symbiotic bacteria levels and a 33-fold reduction in LPS levels over an 18-week period. In the ampicillin-treated and untreated *D. farinae*, the LPS concentrations were measured as 155.6 and 5,135.5 EU/mg, respectively. Previous research found LPS concentrations of 8,740 EU/mg in a Korean *D. farinae* extract and 3,890 EU/mg in a *D. farinae* extract from the United States (8). The protein bands pattern in SDS PAGE analysis of *D. farinae* extract remained unchanged and the concentration of Der f 1 and Der f 2 were not changed by ampicillin treatment.

Then, the *D. farinae* extracts were used to induce asthma in a mouse model (67). The extracts were injected intraperitoneally twice with a 2-week interval, along with alum adjuvant, and challenged intranasally. In our study, we found that the level of lung function, airway inflammation, and serum-specific immunoglobulin did not differ between the mouse asthma models developed using ampicillin-treated *D. farinae* and those developed using ampicillin-untreated *D. farinae*. These findings suggest that *D. farinae* with low bacterial content was sufficient to induce allergic sensitization and immune response. However, another study we conducted demonstrated that administering the extract intranasally at two-day intervals for a total of nine treatments, without the use of an adjuvant, resulted in less pronounced airway inflammation in mice treated with mites that had reduced bacterial levels (68). In this study, the mouse sensitized with the antibiotics-treated mite compared to ampicillin-untreated mite showed less eosinophils in the bronchoalveolar lavage and less immune cell infiltration in lung histopathology. Serum IgE and IgG1 were not different among the groups.

Since bacteria within HDMs can act as adjuvants and exacerbate inflammatory responses, it is crucial to exercise caution when using HDM extract in both clinical and experimental settings. A study has indicated that HDMs can act as carriers of bacteria, leading to the induction of IgE sensitization to bacterial antigens (69). Bacterial IgE sensitization has been associated with different allergic disease conditions, such as the severity of atopic dermatitis and the co-occurrence of rhinitis (70–75). Therefore, reducing bacteria in HDMs would be beneficial, provided that the effectiveness of allergen immunotherapy remains unchanged. Furthermore, standardizing mite production, extract preparation, and controlling bacterial levels are crucial steps to facilitate the development and improvement of diagnostic and therapeutic agents for allergic diseases (76).

## Future research

Further research is needed to better understand the immunological role of symbiotic bacteria in the HDMs. In particular, future studies should be conducted to investigate whether the bacterial components derived from HDM extract exhibit enhanced or diminished efficacy when utilized as immunotherapeutic agents.

Furthermore, additional research is warranted to explore the impact of symbiotic bacteria on the growth of HDMs. Based on our own observations, we have discovered that excessive antibiotic treatment can significantly reduce the growth rate of mites. Further research is required to determine whether this effect is a result of the direct toxicity of antibiotics on HDMs or the consequence of reduced bacterial populations in HDMs.

While our studies indicated that the reduction of bacteria in HDMs did not impact the production of Der f 1 and Der f 2, it is necessary to assess any changes in the expression of other allergens. The composition of the microbiome in storage mites might have an influence on allergen expression (77). Notably, recent findings have suggested that *Cardinium* in *D. farinae* could potentially modulate gene expressions in mites related to immunity and metabolism (78).
